# Use of Different Iron Preparations for Prophylaxis and Effects on Iron Status in Infancy

**DOI:** 10.3390/healthcare12101043

**Published:** 2024-05-18

**Authors:** Merve Tosyalı, Yavuz Demirçelik, Özlem Bağ, Utku Karaarslan, Şule Gökçe, Feyza Koç

**Affiliations:** 1 Department of Pediatrics, Faculty of Medicine, Ege University, Children’s Hospital, 35100 Izmir, Turkey; sule.gokce@yahoo.com (Ş.G.); feyzaumaykoc@yahoo.com (F.K.); 2 Department of Pediatrics, Izmir City Hospital, 35180 Izmir, Turkey; yavuzdemircelik@gmail.com; 3 Department of Pediatrics, Dr. Behçet Uz Pediatric Diseases and Surgery Training and Research Hospital, 35210 Izmir, Turkey; bagozlem78@yahoo.com (Ö.B.); drutkum@gmail.com (U.K.)

**Keywords:** infant, iron, iron deficiency, prophylaxis

## Abstract

**Aim:** To evaluate using different iron preparations for iron deficiency and/or iron deficiency anemia prophylaxis in infants and their iron status. **Methods:** In this study, we retrospectively evaluated the electronic patient records of 651 healthy children aged 9 to 13 months who met the inclusion criteria and who were followed up in pediatric follow-up outpatient clinics between January 2023 and June 2023. **Results:** A total of 651 children with a mean age of 11.2 ± 1.4 months, 54.7% of whom were boys, who met the inclusion criteria were included in the study; 56.5% of the children were using Fe + 3 salt and the others were using Fe + 2 salt, microencapsulated iron, or sucrosomial iron drops. After the fifth month of prophylaxis, when the effects of the iron preparations used on the mean laboratory values were evaluated, it was found that hemoglobin, serum iron, and ferritin levels were lower in sucrosomial iron and microencapsulated iron users compared to other preparations (*p* = 0.001). When statistically pairwise comparisons were made between the groups, hemoglobin and serum iron values were found to be lower in the group using sucrosomial iron compared to the groups using Fe + 2 and Fe + 3 salts (*p* < 0.0001). Hemoglobin and ferritin levels were higher in the group using Fe + 2 salt compared to both sucrosomial iron and microencapsulated iron groups (*p* < 0.0001). When the infants were evaluated according to iron status, it was found that 208 (31.9%) had iron deficiency. Iron deficiency was found to be less in infants of families who defined their economic status as rich and in infants who used iron regularly (*p*-values 0.044 and 0.001, respectively). Iron deficiency/iron deficiency anemia was observed at a higher rate in the group using sucrosomial iron and microencapsulated iron prophylaxis (*p* = 0.001). **Conclusions:** To prevent iron deficiency, it is very important to use appropriate iron preparations for prophylaxis and to feed foods with high iron content. Although we found that families were willing to use different iron preparations other than iron salts for their infants, the results presented herein indicate that the rate of iron deficiency was lower in patients using iron salts. However, randomized controlled studies are needed to determine whether these preparations are effective in iron prophylaxis in infants.

## 1. Introduction

Iron deficiency (ID) is the most common nutritional deficiency and cause of anemia worldwide. It is estimated to affect more than two billion people, especially in developing countries [1,2]. In developing countries, about 40–50% of children suffer from ID [3]. The incidence of ID in children has been reported, ranging from 6.5% to 42% in Turkey [4,5]. Low iron stores (premature birth, low birth weight, insufficient iron stores in the mother, etc.), increased iron requirement, nutrition with foods with low iron content, and low socioeconomic status are among the factors that may lead to ID in early childhood [6,7,8,9,10]. 

ID in early childhood adversely affects brain development by causing delayed myelinization and impaired dopamine metabolism. When iron deficiency is not treated, conditions such as growth–developmental retardation, impaired immunity, decreased IQ level, distractibility, and school failure may occur [6,11,12,13,14]. To prevent ID in early childhood, nutrition with appropriate foods with high iron content and iron supplementation are recommended [15,16,17,18]. However, some sources have reported that effective iron replacement is beneficial to prevent ID/IDA, although some risks of iron use have been reported [19].

The World Health Organization (WHO) recommends the use of iron prophylaxis in countries where the prevalence of anemia is more than 40% or in infants with insufficient dietary iron intake and recommends starting iron prophylaxis in the second month in premature and low-birth-weight infants and the sixth month in term infants [13,20]. The American Academy of Pediatrics recommends iron prophylaxis from the first month of life in premature babies and from the fourth to sixth month of life in term babies who are exclusively breastfed [17]. In our country, iron prophylaxis of 2 mg/kg/day has been recommended from the second month of life in premature babies regardless of nutritional and iron status since 2004, and iron prophylaxis of 10 mg/day is recommended for term infants from the fourth month of life until at least one year of age. The Ministry of Health provides iron (only Fe + 3 salt) preparation to infants free of charge in Family Medicine Centers [13,21]. In addition, ID and iron deficiency anemia (IDA) are evaluated with routine laboratory tests between the 9th and 12th months in infants who have used iron prophylaxis for at least five months [22]. Although the iron prophylaxis program is tried to be carried out effectively as described above, IDA is still common in children under two years of age in our country [5,13,22].

Fe + 2 or Fe + 3 salts are generally recommended for iron prophylaxis [23]. However, approximately 50% of those taking oral iron report gastrointestinal (GI) side effects, which can lead to decreased tolerance and reduced compliance with iron supplementation [24,25]. In recent years, iron preparations with different pharmacokinetics such as microencapsulated iron and sucrosomial iron have been used for ID [26,27]. These preparations are reported not to cause side effects such as GI irritation, often associated with iron salts. In our country, these preparations can be purchased by families and used for iron prophylaxis. However, sufficient studies are comparing the effectiveness of these preparations in preventing ID. This study aimed to evaluate the use of different iron preparations with different pharmacokinetics, such as microencapsulated iron and sucrosomial iron prophylaxis, in addition to the commonly used Fe + 3 or Fe + 2 salts, and their effect on iron levels in infants.

## 2. Method

### 2.1. Data

In this study, electronic patient records of healthy children who live in city centers and are followed up in outpatient clinics were retrospectively evaluated. The records of 1100 children admitted between January 2023 and June 2023 were cross-sectionally analyzed. The study was conducted according to the guidelines of the Declaration of Helsinki. The approval of the study was obtained from the Ege University Medical Research Ethics Committee (23-3.1T/11). Informed consent was obtained from all subjects involved in the study.

#### 2.1.1. Inclusion Criteria

– Mature infants born at 37 weeks of gestation (>36 + 1 gestational weeks) or later, and premature infants born between 36 + 1 and 36 + 6 weeks of gestation;
– With a history of singleton births;
– Exclusively breastfed for the first six months;
– Have received iron prophylaxis at an appropriate dose for at least five months after starting iron prophylaxis in the fourth month;
– No concomitant chronic disease;
– Healthy infants who underwent routine blood evaluation for screening for iron deficiency anemia between 9 and 13 months of age were included in the study.

#### 2.1.2. Exclusion Criteria

– Premature birth (<36 + 1 gestational weeks);
– With a history of multiple gestational births;
– Intrauterine growth retardation, chronic illness, history of prolonged hospitalization or acute infection;
– Infants who did not meet the inclusion criteria or whose data were missing in the electronic patient file were excluded from the study.

### 2.2. Variables

In addition to the general characteristics of the cases, such as age, gender, birth week, weight, sociodemographic characteristics of the families (education, employment, and economic status of the parents), the mother’s vegetarianism and history of anemia during pregnancy, the iron preparations used by the children, whether they used them regularly, and the use of red meat products were evaluated. Blood hemoglobin (Hb), red blood cell (RBC), mean erythrocyte volume (MCV), serum iron (SI), iron-binding capacity (TIBC), and ferritin parameters were recorded for anemia and iron status.

### 2.3. Definitions Description

The infants included in the study were evaluated in 4 groups as Fe + 3 salt, Fe + 2 salt, microencapsulated iron, and sucrosomial iron, according to the iron preparations they used. The appropriate dose was considered to be 10 mg/day for Fe + 2 and Fe + 3 salts, and one cc (8 and 7 mg/day, respectively) for microencapsulated iron and sucrosomial iron preparations as written in the package insert [13,21,28]. The use of iron at an appropriate dose more than five days a week was defined as regular iron use. According to the infants’ feeding history, red meat consumption more than 1–2 days a week was defined as “adequate meat consumption” [29].

The children included in the study were evaluated in 2 groups as normal, ID, and/or IDA in terms of iron status. SI < 30 mg/dL and/or Ferritin < 12 microgram/L was defined as ID and SI < 30 mg/dL and/or Ferritin < 12 microgram/L, Hb < 11 g/dL was defined as IDA as stated in the WHO guidelines (for infants aged 6–59 months) [20,30]. While evaluating the economic status, parents were asked, “How do you perceive your economic status?”. The answers were recorded as normal, rich, and poor.

### 2.4. Statistics

The statistical analysis was performed by using the SPSS 18.0 package program (PASW Statistics for Windows, Version 18.0, Chicago, IL, USA). The numerical data were presented as mean ± standard deviation or median (interquartile range) and rates as percentages. In the evaluation of numerical data with normal distribution between the groups, one-way analysis of variance test was used, and as a post hoc method, Tamhane’s T2 test was conducted. For the comparison of numerical data without normal distribution, Kruskal–Wallis test was used, and as a post hoc method, Mann–Whitney U-test was used. For the comparison of rates in the groups, χ^2^ test was conducted. A *p*-value of <0.05 was accepted as significant.

## 3. Results

A total of 651 children with a mean age of 11.2 ± 1.4 months, 54.7% of whom were boys, who met the criteria were included in the study. The general characteristics of the children and families included in the study are shown in Table 1. 45.3% (n = 295) of the mothers and 47.6% (n = 310) of the fathers were university graduates; 485 (74.5%) of the families stated that they perceived their economic status as “normal”. 

While 368 (56.5%) of the children included in the study were using Fe + 3 salt drops provided free of charge by the Ministry of Health, the others were using Fe + 2 salt, microencapsulated iron, sucrosomial iron [76 (11.7%), 48 (7.4%), and 159 (24.4%), respectively] iron drops purchased by their own means. It was determined that 562 (86.3%) of the infants used iron drops regularly. Table 2 shows the demographic characteristics of the children according to the iron preparation they used.

According to the preparation used, the educational level of the mother, father, and working status of the mother were found to be statistically significantly different (*p* < 0.05). It was determined that the rate of using microencapsulated or sucrosomial iron drops was higher when the parents had high school or higher education levels and the mothers were employed. No statistically significant difference was found when other demographic characteristics such as age, gender, weight, time of birth, economic status, and regular use were analyzed (Table 2).

The children included in the study were compared according to the laboratory analyses performed for the evaluation of anemia (RBC, MCV, Hb, SI, ferritin, and TIBC) and iron preparations used between 9 and 13 months (Table 3). Hb, SI, and ferritin levels of children who used sucrosomial iron were lower than the other groups (*p*-values 0.001, 0.007, and 0.03, respectively). When statistically pairwise comparisons were made between the groups, Hb and SI values were found to be lower in the group using sucrosomial iron compared to the groups using Fe + 2 and Fe + 3 salts (*p* < 0.0001) (Table 3). Hb and ferritin levels were higher in the group using Fe + 2 salt compared to both sucrosomial iron and microencapsulated iron groups (*p* < 0.0001). In addition, it was found that Hb level was higher in the group using Fe + 2 salt than in the group using Fe + 3 salt (*p* < 0.0001).

**Figure 1:**
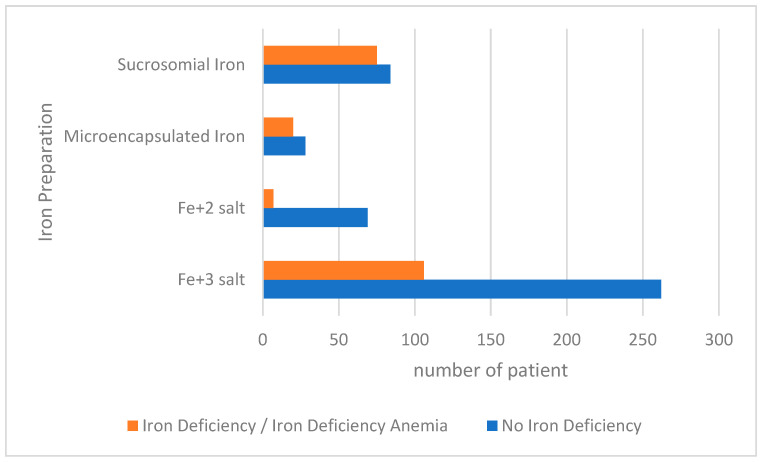
Effect of groups organized according to the prophylactic iron preparation used on iron deficiency.

When the infants were evaluated according to iron status, it was found that 208 (31.9%) had ID. Among the cases with ID, 38 (5.8%) had only ID, and 170 (26.1%) had IDA. Factors that may affect the iron status of children were evaluated and are presented in Table 4. A statistically significant difference was found between the perceived economic status as rich and regular iron use and ID. ID was found to be less in infants of families who defined their economic status as rich and in infants who used iron regularly (*p*-values 0.044 and 0.001, respectively) (Table 4). When ID status was evaluated according to the iron preparations used by the babies, it was determined that 3.4% (n = 7) of the babies in the ID/IDA group used Fe + 2 salt, 51% (n = 106) used Fe + 3 salt, 36.1% (n = 75) used sucrosomial iron, and 9.6% (n = 20) used microencapsulated iron (Table 4). The percentage of ID/IDA was statistically different between the groups using different iron preparations (*p* = 0.001). When statistically pairwise comparisons were made between the groups, it was determined that the groups using Fe + 2 salt had less ID/IDA than the groups using Fe + 3 salt, microencapsulated iron, and sucrosomial iron (*p* < 0.0008). It was found that ID/IDA was more common in sucrosomial iron users compared to other groups (*p* < 0.0008). When the ID/IDA ratios of the groups organized according to the prophylactic iron preparation used were evaluated, the groups with the highest ID/IDA ratios were sucrosomial iron and microencapsulated iron groups, respectively (Figure 1). The parents’ educational level, employment status, and adequate meat consumption were not statistically significantly different in the group with ID/IDA (Table 4).

## 4. Discussion

It is known that ID is the most common nutritional deficiency worldwide. In our country, the “Iron Like Turkey” program has been carried out since 2004. Free iron (Fe + 3) support is provided to premature babies from the second month and mature babies from the fourth month until the age of one year. However, sometimes families can buy and use other iron preparations sold for a fee at their request or the recommendation of healthcare professionals (private doctors, pharmacists, nurses, etc.). Although Fe + 2 drops are the best known among other iron preparations, sucrosomial iron or microencapsulated iron drops have also been used in recent years. Our study is critical because it is the first study to investigate the effect of different iron preparations used in prophylaxis on iron status in infants.

Fe + 2 or Fe + 3 salts are generally recommended for iron prophylaxis [23]. According to the current literature, Fe + 2 salt has been reported to be more effective than Fe + 3 salt in the treatment of ID [31]. This is because Fe + 2 ions taken from an empty stomach are more easily absorbed by the intestines and rapidly metabolized in the cell. Inside the cell, Fe + 2 ions are oxidized to Fe + 3 ions and transported between cells by iron transport proteins such as transferrin and used for hemoglobin synthesis in hematopoietic cells. The rapid absorption and intracellular utilization of Fe + 2 ions provide faster relief of ID symptoms. In our study, it was found that infants mostly used Fe + 3 salt (43.5%) provided free of charge. The most commonly used iron salts for iron prophylaxis (Fe + 2 and Fe + 3 salts) may sometimes not be preferred by families because of side effects such as nausea, vomiting, and abdominal pain [24,25,32]. In our study, it was found that different iron preparations such as sucrosomial iron and microencapsulated iron, which have been used in recent years and reported to have fewer GI side effects, were preferred by families. However, the reasons why families preferred these preparations were not questioned.

When Hb, SI, and ferritin values of the infants were analyzed according to the iron preparations used, it was found that the group using Fe + 2 salt had the highest Hb, SI, and ferritin values, while those using sucrosomial iron had lower Hb, SI, and ferritin values compared to the others.

In addition, in our study, ID/IDA was found to be less common in infants using Fe + 2 salt and most common in those using sucrosomial and then microencapsulated iron (Table 4, Figure 1). It is known that commonly used iron salts prevent ID and at which doses they should be used. In addition, Fe + 2 salt has been reported to be more effective in the prevention and treatment of ID [33,34,35]. The results of our study also support this information. Studies on sucrosomial or microencapsulated iron preparations prepared in different formulations are limited [24,25,36,37,38,39,40]. No randomized controlled studies are reporting whether these preparations are effective in preventing ID in children and what the appropriate dose should be. Therefore, families and physicians should be aware of the iron drops used. Caution should be exercised in the use of iron preparations, which are reported to be effective by different mechanisms.

In our study, it was found that 31.9% of the infants had ID/IDA. In previous studies in our country, ID in childhood has been reported at different rates including 6.5–42% [4,5]. In other studies, it has been reported that ID continues to be observed even if babies are given prophylaxis [5,13]. In addition to prophylaxis to prevent ID, it is very important to give foods with high iron content to infants after the 6th month [41]. In our study, no statistically significant difference was found between the iron status of infants and adequate meat consumption. However, feeding of infants with other foods with high iron content other than meat was not questioned. In addition to iron replacement, the main goal should be to ensure that infants are fed foods with high iron content or to increase the amount of iron in foods (e.g., by supplementing the iron content of milk, flour, etc.).

## 5. Conclusions

In our study, it was found that families were willing to use different iron preparations other than iron salts for their infants. However, randomized controlled studies are needed to determine whether these preparations are effective in iron prophylaxis in infants. In our country, the efficacy of the iron preparation used free of charge in infants in the first year of life needs to be reviewed. It was found that the groups using Fe + 2 salt had less ID/IDA than the groups using Fe + 3 salt, microencapsulated iron, and sucrosomial iron. When the ID/IDA ratios of the groups organized according to the prophylactic iron preparation used were evaluated, the groups with the highest ID/IDA ratios were sucrosomial iron and microencapsulated iron groups, respectively. In addition, it is thought to be very important to check whether iron levels are effective when iron preparations other than iron salts are used in prophylaxis. As a result of this evaluation, when ID/IDA is detected, it must be treated. To prevent ID, prophylactic use of appropriate iron preparations and a diet high in iron is very important.

## 6. Limitations

Our study is a cross-sectional and retrospective study, and the number of cases in the determined groups is small. Information such as sociodemographic characteristics, iron preparations used, regular and irregular use, and dose information were obtained from electronic files.

The nutritional characteristics and iron replacement status of the mothers in this pregnancy and the nutritional characteristics of the infants could not be questioned in detail. Because of the small sample size, the results obtained cannot be generalized to the general population.

## Figures and Tables

**Table 1:** Demographic characteristics (n, %).

Age (month) *	11.2 ± 1.4
Weight (gram) *	9939 ± 1229
Height (cm) *	74.7 ± 3.7
Gender	
Male	356 (54.7)
Female	295 (45.3)
Time of birth	
Mature	544 (83.6)
Premature	107 (16.4)
Mother’s education	
1–8 years	154 (23.6)
9–12 years	202 (31)
University	295 (45.3)
Father’s education	
1–8 years	80 (12.3)
9–12 years	261 (40.1)
University	310 (47.6)
Occupational status of mother	
Housewife	302 (46.4)
Working	349 (53.6)
Occupational status of father	
Unemployed	12 (1.8)
Working	639 (98.2)
Family’s financial situation	
Poor	139 (21.4)
Normal	485 (74.5)
Rich	27 (4.1)

* mean ± SD.

**Table 2:** Comparison of iron preparations used by infants and their demographic characteristics (n, %).

	Fe + 3 Salt n = 368	Fe + 2 Salt n = 76	Microencapsulated Iron n = 48	Sucrosomial Iron n = 159	*p*
Age (month) *	11.2 ± 0.73	11.2 ± 0.16	11.1 ± 0.22	11.1 ± 0.10	0.29
Weight (gram) *	9986 ± 1236	9803 ± 1190	9836 ± 1267	9927 ± 1223	0.6
Gender					0.93
Male	200 (54.3)	40 (52.6)	28 (58.3)	88 (55.3)	
Female	168 (45.7)	36 (47.4)	20 (41.7)	71 (44.7)	
Time of birth					0.9
Mature	307 (83.4)	65 (85.5)	41 (85.4)	131 (82.4)	
Premature	61 (16.6)	11 (14.5)	7 (14.6)	28 (17.6)	
Mother’s education					0.015
1–8 years	89 (24.2)	20 (26.3)	10 (20.8)	35 (22)	
9–12 years	127 (34.5)	29 (38.2)	7 (14.6)	39 (24.5)	
University	152 (41.3)	27 (35.5)	31 (64.6)	85 (53.5)	
Father’s education					0.001
1–8 years	54 (14.6)	8 (10.5)	6 (12.5)	12 (7.5)	
9–12 years	162 (44)	34 (44.7)	10 (20.8)	55 (34.6)	
University	152 (41.3)	34 (44.7)	32 (66.7)	92 (57.9)	
Occupational status of mother					0.001
Housewife	187 (50.8)	42 (55.3)	22 (45.8)	51 (32.1)	
Working	181 (49.2)	34 (44.7)	26 (54.2)	108 (67.9)	
Occupational status of father					0.23
Unemployed	8 (2.2)	3 (3.9)	0	1 (0.6)	
Working	360 (97.8)	73 (96.1)	48 (100)	158 (99.4)	
Family’s financial situation					0.47
Poor	72 (19.6)	13 (17.1)	11 (22.9)	43 (27)	
Normal	281 (76.4)	59 (77.6)	34 (70.8)	111 (69.8)	
Rich	15 (4.1)	4 (5.3)	3 (6.3)	5 (3.1)	
Iron usage situation					0.59
Regular	318 (86.4)	62 (81.6)	42 (87.5)	140 (88.1)	
Irregular	50 (13.6)	14 (18.4)	6 (12.5)	19 (11.9)	

* mean ± SD.

**Table 3:** Average laboratory values of the iron preparation groups used.

	Fe + 3 Salt n = 368	Fe + 2 Salt n = 76	Microencapsulated Iron n = 48	Sucrosomial Iron n = 159	*p*
RBC	4.7 ± 0.4	4.6 ± 0.3	4.7 ± 0.3	4.5 ± 0.3	0.001
MCV	76.6 ± 4.3	76.9 ± 4.9	76.9 ± 5.1	75.4 ± 4	0.002
Hb	11.5 ± 0.9	11.9 ± 0.7	11.4 ± 1.2	10.9 ± 1.1	0.001
SI	52.6 ± 24.9	55.4 ± 23.1	51.2 ± 23.7	46.3 ± 22.7	0.007
Ferritin	32.3 ± 22.5	31.8 ± 15.8	27.6 ± 18.5	24.5 ± 15.6	0.03
TIBC	358 ± 56.7	360.5 ± 81.8	368.9 ± 74	381.7 ± 67.5	0.11

Mean ± SD, RBC: red blood cell; MCV: mean corpuscular volume; Hb: hemoglobin; SI: serum iron, TIBC: total iron-binding capacity.

**Table 4:** Factors affecting iron deficiency (n, %).

	No Iron Deficiency n = 443	Iron Deficiency and/or Iron Deficiency Anemia n = 208	*p*
Age (mean ± SD)	11.3 ± 1.4	10.9 ± 1.4	0.004
Gender			0.33
Male	248 (56)	108 (51)	
Female	195 (44)	100 (48.1)	
Time of birth			0.12
Mature	377 (85)	167 (80.3)	
Premature	66 (15)	41 (19.7)	
Mother’s education			0.17
1–8 years	93 (21.3)	13 (34.2)	
9–12 years	140 (32)	6 (15.8)	
University	204 (46.7)	19 (50)	
Father’s education			0.42
1–8 years	48 (10.9)	6 (15.8)	
9–12 years	172 (39)	18 (47.4)	
University	221 (50.1)	14 (36.8)	
Occupational status of mother			0.51
Housewife	201 (45.6)	15 (39.5)	
Working	240 (54.4)	23 (60.5)	
Occupational status of father			0.13
Unemployed	6 (1.4)	0	
Working	435 (98.6)	38 (100)	
Family’s financial situation			0.044
Poor	89 (20.1)	50 (24)	
Normal	333 (75.2)	152 (73.1)	
Rich	21 (4.7)	6 (2.9)	
Mother’s status as a vegetarian			0.19
Yes	1 (0.2)	2 (1)	
No	441(99.8)	205 (99)	
The mother has anemia during pregnancy			0.68
Yes	111 (25.1)	49 (23.6)	
No	332 (74.9)	159 (76.4)	
Meat consumption			0.41
Sufficient	405 (91.4)	186 (89.4)	
Insufficient	38 (8.6)	22 (10.6)	
Iron preparation groups			0.001
Fe + 3 salt	262 (59.1)	106 (51)	
Fe + 2 salt	69 (15.6)	7 (3.4)	
Microencapsulated iron	28 (6.3)	20 (9.6)	
Sucrosomial iron	84 (19)	75 (36.1)	
Iron usage situations			0.001
Regular	408 (92.1)	154 (74)	
Irregular	35 (79)	54 (26)	

## Data Availability

Data are contained within the article.
